# Toward a Hybrid Passive BCI for the Modulation of Sustained Attention Using EEG and fNIRS

**DOI:** 10.3389/fnhum.2019.00393

**Published:** 2019-11-06

**Authors:** Alexander J. Karran, Théophile Demazure, Pierre-Majorique Leger, Elise Labonte-LeMoyne, Sylvain Senecal, Marc Fredette, Gilbert Babin

**Affiliations:** HEC Montréal, Université de Montréal, Montréal, QC, Canada

**Keywords:** BCI (Brain Computer Interface), EEG, fNIRS (functional near infrared spectroscopy), HCI (human computer interaction), sustained attention, wavelet coherence

## Abstract

We report results of a study that utilizes a BCI to drive an interactive interface countermeasure that allows users to self-regulate sustained attention while performing an ecologically valid, long-duration business logistics task. An engagement index derived from EEG signals was used to drive the BCI while fNIRS measured hemodynamic activity for the duration of the task. Participants (*n* = 30) were split into three groups (1) no countermeasures (NOCM), (2) continuous countermeasures (CCM), and (3) event synchronized, level-dependent countermeasures (ECM). We hypothesized that the ability to self-regulate sustained attention through a neurofeedback mechanism would result in greater task engagement, decreased error rate and improved task performance. Data were analyzed by wavelet coherence analysis, statistical analysis, performance metrics and self-assessed cognitive workload via RAW-TLX. We found that when the BCI was used to deliver continuous interface countermeasures (CCM), task performance was moderately enhanced in terms of total 14,785 (σ = 423) and estimated missed sales 7.46% (σ = 1.76) when compared to the NOCM 14,529 (σ = 510), 9.79% (σ = 2.75), and the ECM 14,180 (σ = 875), 9.62% (σ = 4.91) groups. An “actions per minute” (APM) metric was used to determine interface interaction activity which showed that overall the CCM and ECM groups had a higher APM of 3.460 (*SE* = 0.140) and 3.317 (*SE* = 0.139) respectively when compared with the NOCM group 2.65 (*SE* = 0.097). Statistical analysis showed a significant difference between ECM - NOCM and CCM - NOCM (*p* < 0.001) groups, but no significant difference between the ECM – CCM groups. Analysis of the RAW-TLX scores showed that the CCM group had lowest total score 7.27 (σ = 3.1) when compared with the ECM 9.7 (σ = 3.3) and NOCM 9.2 (σ = 3.4) groups. No statistical difference was found between the RAW-TLX or the subscales, except for self-perceived performance (*p* < 0.028) comparing the CCM and ECM groups. The results suggest that providing a means to self-regulate sustained attention has the potential to keep operators engaged over long periods, and moderately increase on-task performance while decreasing on-task error.

## Introduction

The current and predicted pace of digital transformation, through the application of artificial intelligence (AI) and robotic process automation (RPA) is changing many of the characteristics of work, leisure, travel, and other human activities. These transformations have increased the capability and availability of computer hardware and software to carry out physical and cognitive labor in addition to reducing these costs. These factors in combination with fiscal policy are now driving the acceleration and adoption of automation within organizations and their workplaces, replacing human labor and fundamentally changing the way in which we interact with technology (Autor, 2015).

As a way of potentially combating the complete automation of human labor, there is currently a growing interest in industrial and research approaches which include “human in the loop” technological augmentations and solutions. In the area of physical capacity augmentation recent developments by Ford Motors (Reid et al., 2017) presents a perfect example of a human-in-the-loop solution in its rollout of exoskeletons to augment the physical capabilities of their skilled workforce, enhancing the strength, endurance, and mobility of workers during overhead tasks. This innovative use of human-machine technological augmentation in the domain of physical labor represents a significant first step in human capacity augmentation. However, the rapid adoption of RPA into many business and enterprise models is now automating cognitive tasks once thought of as traditionally human (van der Aalst et al., 2018). These tasks, such as those requiring the need for judgment, pattern recognition and the ability to communicate effectively, are now amenable to RPA through the application of machine learning (Wilson, 2005). Moreover, RPA, through process efficiency gains, has created large subsets of tasks that require human intervention only at key points in a process operation. Thus, RPA is changing the position of the human-in-the-loop (who formerly instigated and completed tasks) from being a primary agent to a form of “middleman,” who monitors complex processes and then provides process validation through decision making (Lacity and Willcocks, 2018).

While automation has increased productivity through a reduction in information-processing and cognitive load, it has also lead to decreased on-task safety and increased incidents during safety-critical operations due to monitoring error. Incidents triggered by on-task monitoring surprise, are often the result of a decrease in operator vigilance and sustained attention (Parasuraman et al., 1996; Miller and Parasuraman, 2003; De Boer and Dekker, 2017). In the new automated workplace, the ability to maintain a vigilant state, characterized as a process of sustained attention or tonic alertness, may become a valuable asset. A vigilant state implies both a high degree of physiological arousal and a high level of sustained cognitive performance. Research in human factors has identified several characteristics necessary to maintain a state of vigilance such as sustained attention, signal detection, staying alert, target identification and maintaining performance over time through task engagement (Donald, 2001; Adams and Adams, 2002; Prinzel et al., 2002). However, the terms vigilance, sustained attention, and mental workload are used interchangeably within the literature, as facets of the same phenomena. For the purposes of the research presented in this manuscript, we utilize the term sustained attention (SA) to cover tonic alertness, attention, and the vigilance decrement as defined by Oken et al. (2006).

### The Brain–Computer Interface (BCI): A Tool to Augment Human Capacities

Within the past decade, BCIs have fast become tools to augment cognitive capacities and overcome physical impairments. Improvements in sensor technologies and brain-activity classification have shown BCIs to be both a useful assistive technology and a general interface technology for human–machine systems (Zander and Kothe, 2011). BCIs, defined as “a device that reads voluntary changes in brain activity, then translates these signals into a message or command in real-time” (Guger et al., 2015) are systems which utilize the neurophysiological data of the user as an input to a computer system, which then performs actions to assist or provide feedback to the user. A BCI relies upon signals derived from the brain, and commonly these signals are monitored or recorded using electroencephalography (EEG), through the placement of electrodes on the surface of the head.

In the medical field BCIs have been applied to assist with the control of various prosthesis (Hong and Khan, 2017), such as robotic arm control for users with spinal cord injury (Nicolas-Alonso and Gomez-Gil, 2012), or as additional input to the classic controllers for wheelchair control (Carlson and del Millan, 2013). In addition, BCIs are seeing significant application outside of the medical domain, such as within information system research to provide decision support (Mai et al., 2017) and in user experience research and entertainment (Muñoz et al., 2017). Another growing area for BCIs is mental workload estimation, exploring the effects of mental workload and fatigue upon the P300 response (used for word spell BCI) and the alpha-theta EEG bands (Käthner et al., 2014). In this regard, there is currently a movement within the BCI community to integrate other signal types into “hybrid BCIs” (Pfurtscheller et al., 2010) to increase the granularity of the monitored response.

### Hybrid BCI for Cognitive State Estimation

Foremost amongst these techniques is functional near-infrared spectroscopy (fNIRS); an optical neuroimaging technique which can distinguish concentration changes of oxygenated and deoxygenated hemoglobin (HbO and HbR) on the outer cortical layers of the brain (e.g., Derosière et al., 2013 for a review). This technique has been used to measure changes in mental workload (MWL) with regards to stress and anxiety when performing imaging tasks (Alsuraykh et al., 2018). Boyer et al. (2015) investigated changes in MWL using a long duration supervisory monitoring control task. They found that fNIRS was feasible for use in long-duration tasks; that the hemodynamic response diminished toward the middle of the task; that fNIRS was unable to detect changes in workload, but rather reflected temporal changes in task event onsets, which could potentially be used to auto-adapt a system when operators are in a reduced attentional state. In work exploring the combination of fNIRS and EEG for MWL classification using machine learning approaches, Hirshfield et al. (2009) found that there were several technical issues that affect NIRS and EEG when used concurrently such as sensor placement and the potential for fNIRS light sources to introduce noise into the EEG signal. However, they did find that fNIRS responses were classifiable with moderate levels of accuracy for MWL states, showing that fNIRS signal has potential when considering real-time detection and auto-adaptive systems.

In a study investigating the use of fNIRS as an objective concurrent measure of MWL in usability testing, Lukanov et al. (2016) found that fNIRS provided a reliable measure of MWL in usability testing and that this reliability would translate from psychology tasks to more ecologically valid tasks. The same level of reliability and was reported in work concerned with realistic human–computer interaction (HCI) contexts (Maior et al., 2015) which found that fNIRS was able to distinguish between cognitive and rest states in both verbal and spatial task. In a study to determine if fNIRS could distinguish differences in cognitive state associated with difference in visual design, Peck et al. (2013) found that fNIRS provided a robust measure of mental effort associated with visual design when measured from the prefrontal cortex (PFC). They suggested that fNIRS may prove most effective at measuring cognitive states when used in more complex analytical interactions. In a similar HCI context, Maior et al. (2014) utilized fNIRS to measure how different forms of verbalizations affect cognitive workload, using a think-aloud protocol (TAP) experiment to induce activity in cortical areas associated with verbal working memory. Using a combination of NASA-TLX and fNIRS, they found that verbalization did not affect the fNIRS measure in terms of artifacts and that fNIRS provided a clear indication of participants MWL while completing the task and that fNIRS can be used to determine MWL objectively during tasks if verbalizations are task-related. Other work by Khan et al. (2014) explored hybrid NIRs-EEG for decoding four movement directions, they induced changes in HbO through mental arithmetic tasks as forward and backward directional signals and changes induced in EEG through a hand tapping task as left and right directional signals. They reported classification accuracies using this method in excess of 80%. In later work, Hong et al. (2018) investigated the use of a hybrid BCI for by patients with locked in syndrome. They posited the use of vector phase analysis as a classifier of brain behavior, combining feature extraction and classification methods into a single framework which may prove to be a promising solution for brain therapies for those with locked in syndrome.

Generally, the benefit of integrating EEG and fNIRS signals into a BCI is to take advantage of the strengths of each measure to drive the feedback mechanism. EEG offers good temporal resolution (∼5 ms), whereas fNIRS offers better spatial resolution (2–3 cm) and has been reported to be more robust to artifacts (Scholkmann et al., 2014). NIRS has been used successfully to investigate mental workload assessment and vigilance in ecologically valid contexts, such as with UAV operators (Ayaz et al., 2012), driving vehicles (Yoshino et al., 2013), outdoor navigation using wearable augmented reality displays (McKendrick et al., 2016), investigating mental workload in a long duration supervisory control task (Boyer et al., 2015) and measuring workload in real-time during an *n*-back cognitive and working memory task (Coffey et al., 2012). The combination of fNIRS and EEG for BCI has been utilized in a number of medical studies to enhance performance in sensory-motor rhythm tasks (Fazli et al., 2012), detecting movement commands (Khan and Hong, 2017) and for the classification of auditory and visual perception processes (Putze et al., 2014).

### Augmenting Sustained Attention Using BCI

At present, there are few examples of a BCI that modulates a user’s level of sustained attention. The first of these was created by Pope et al. (1995), who developed a task engagement index derived from brain activity to drive an adaptive system. The goal of the system was to modulate the cognitive workload of the user to maintain an optimal level of task engagement and thus enhance task performance. The same approach was taken by Parasuraman et al. (1996) to study the effect of adaptive task allocation during a monitoring task. This “engagement index” has been used in a number of more recent studies to modulate signal event rates in real-time during a vigilance task (Mikulka et al., 2002; Freeman et al., 2004) and adapt the difficulty of a video game in real-time (Ewing et al., 2016). Findings from these studies showed that the engagement index could prove volatile when used in real-time without some form of a computational classifier, which considers how a user’s level of measured engagement evolves over time. Consequently, the difficulty level of the game would shift too rapidly, inducing phases where cognitive demand was overloaded.

In this study, we investigate the effects of a BCI developed to allow users to modulate their level of sustained attention over a long duration task (Demazure et al., 2019). We developed an ecologically valid long-duration business task designed to induce a decrement in sustained attention and vigilance, which employs an information dashboard that requires long monitoring and short decision cycles. To monitor sustained attention, we developed a BCI using measures of EEG to create an engagement index, which runs in real-time and drives a simple interface countermeasure based upon the current estimated level of sustained attention. In addition, we recorded fNIRS data concurrently to observe the effect of the BCI upon cerebral blood flow and make a *one to many* inference between the level of sustained attention and measures of task engagement. Previous research has shown that measures of task engagement and cerebral blood flow velocity can provide a meaningful diagnostic when monitoring sustained attention (Matthews et al., 2010). Furthermore, evidence for this inferential link has been shown in fMRI studies using a sustained attention task (Forster et al., 2015). However, for this study we are not seeking to integrate measures of cerebral blood flow as features within the BCI architecture, but rather to use fNIRS as a measure to monitor behavioral changes for users of the BCI. Future research will utilize the fNIRS data to derive features for a full hybrid BCI that integrates both EEG and fNIRS to monitor and drive behavioral changes.

In this work, we use the engagement index proposed by Pope and colleagues to provide both a proxy for the level of sustained attention during task completion and as input for the BCI. The BCI integrates a novel computational classifier which is trained initially during a brief calibration task, and then continuously adapts to changes in threshold values as the task is performed in order to reduce the previously reported volitivity of the measure. Brain activity was recorded continuously using fNIRS from frontal and occipital areas and utilized in the analysis to determine significant brain activity during each task cycle. This data was then coupled with the engagement index data to produce a wavelet coherence analysis (Verdière et al., 2018), to provide analysis and visualizations of coherence activity between the BCI and measured fNIRS response. Furthermore, we position the observed effect(s) within the theoretical compensatory control, cognitive-energetical framework proposed by Hockey (1997).

## Materials and Methods

### Participants

Thirty participants (11 female; ages 18–43, μ 24) taken from our institution’s panel of Business School participants took part. Participants were screened on the basis of good health, average hair density, and normal or corrected to normal vision. All participants had some familiarity with the type of task used for the experiment, having completed business courses presenting tasks of a similar nature during their program of study. Participants were provided with a mouse and keyboard and sat approximately 80 cm in front of a 24′′ computer screen within a room with an integrated Faraday cage and asked to keep any unnecessary head movements to a minimum. The typical total duration of a participant’s session (informed consent, sensor application, calibration, business task, questionnaire, and de-briefing) was 3 h. All participants provided signed consent in line with the University’s research ethics committee and compensated $50 CAD. Due to the variable ergonomic factors related to the wearing of fNIRS sensor technology for long durations, all participants were informed that they could leave the experiment if at any time levels of physical discomfort exceeded bearable levels.

### Experimental Design

**FIGURE 1:**
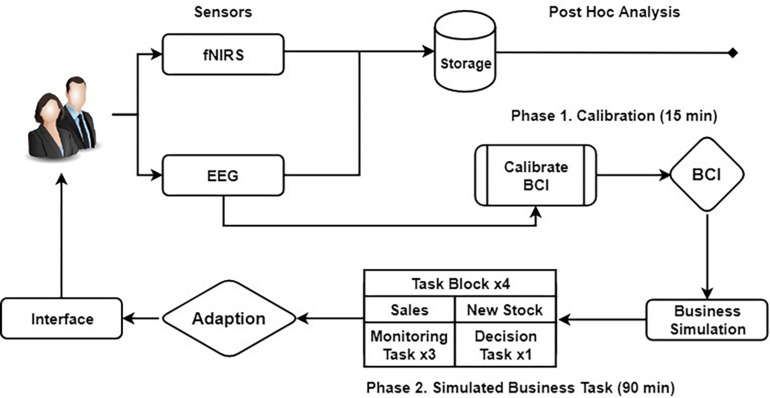
The complete experimental schema and neurofeedback loop visualization.

The experiment used a between-subjects design with three groups. The task was split into two phases: Calibration and Simulated Business Task. Phase 1 consisted of a 15-min task used to calibrate the BCI for real-time operation. Phase 2 consisted of a 90-min business logistics task performed in real-time, utilizing the BCI. Figure 1 illustrates the experimental schema. Our experimental task was adapted from Pattyn et al. (2008) who used a similar task and timing protocol for investigating the effects of the vigilance decrement upon task performance. However, our goal here was to utilize participant vigilance behavior to derive a number of initial threshold values ranging from low-to-high to calibrate the BCI before real-time operation.

#### Phase One: Calibration Task

**FIGURE 2:**
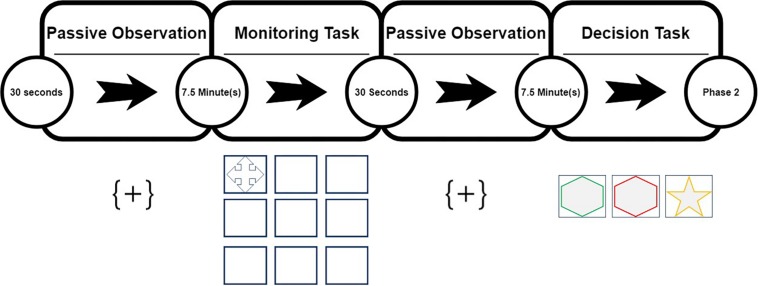
Phase 1 calibration task.

The calibration phase (15 min) was composed of a 30 s baseline; i.e., passive observation of a still image, followed by two active tasks lasting 7.5 min (Figure 2). The first task (monitoring) involved participants visually monitoring nine squares displayed upon a computer screen. Within 1 square, a cross is presented every 5s, the appearance of the cross is preceded by a preamble image lasting 2 s, followed by the presentation of the cross itself displayed for 3 s. Thus, every 5 s the cross appears to move in a clockwise direction. Participants were required to signal they observed the change in position by pressing the number 1 on the keypad provided.

Task 2 (decision) was preceded by another 30 s baseline, after which the second task began. In this task, three simple shapes were displayed, with the appearance of the shapes preceded by a preamble image lasting 2 s with the shapes displayed for 3 s. Each shape is outlined in one of three colors (red, amber, green) of which red was the target. With the exception of the red outline, the shapes and outlines are displayed in random order for five iterations. On the 6th iteration, the target outline is displayed upon one shape at random. Participants were asked to press the number two key on a keyboard number pad when the target outline (in red) was displayed, otherwise to press the one key.

#### Phase 2 Simulated Business Task

**FIGURE 3:**
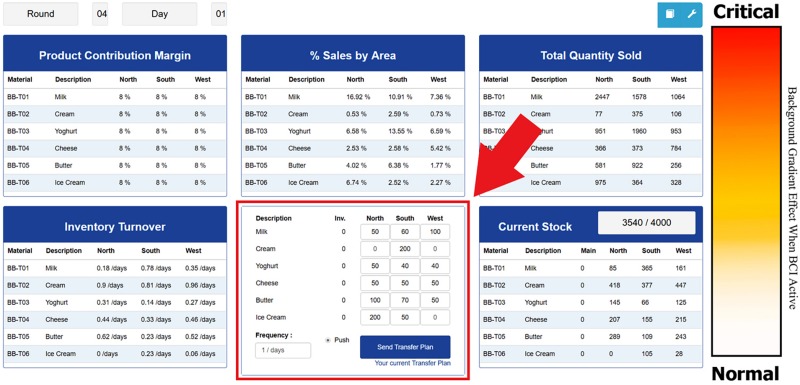
The decision-monitoring business task interface with task interface countermeasure, **(Left)** arrow denotes interactive decision console; **(Right)** the color gradient feedback schema, red to amber = very low to low sustained attention, white = moderate to high level of sustained attention; interface background changes color in accordance with level of sustained attention.

The simulated business task is a continuous task that lasted 90 min and consisted of a 10-min practice period in which participants became familiar with the task environment (Figure 3) followed by an 80-min simulated business logistics task. The business logistics task was made up of 2 distinct task types – decision tasks and monitoring tasks.

• A decision task requires the participant to interact with the user interface to create and input a stock transfer plan consisting of the number of consumables for sale within each business zone.
• The monitoring task requires the participant to observe the effect of the stock transfer plan upon sales for three business zones in order to create a strategy -mentally- for the next stock transfer plan. This plan is then input during the next decision task.
• Task completion follows a scripted order based upon a timeline of events with 4 min to complete a decision task and 16 min to complete a monitoring task.

The simulated business task was created using a simulated enterprise system called ERPsim (Léger, 2006), ERPsim simulates a real-life organization and allows for complete control of all the elements within the task. The task interface is organized to represent a standard business “dashboard” (see Figure 3), consisting of areas to monitor sales and a decision console (marked in red) which allows participants to make logistics decisions by directing stock levels to each of the “areas” requiring replenishment through direct interaction with the console. Participants interact with this interface using a mouse and keyboard number pad to input their purchasing and sales strategy during decision tasks. Values to be monitored are updated in real-time and displayed on the monitor in the interface elements.

**FIGURE 4:**
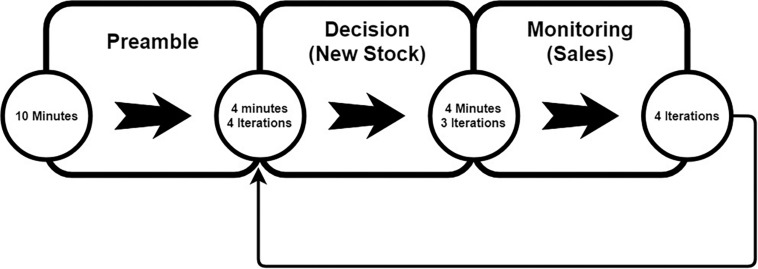
Simulated business task timeline.

The two types of task are presented in cycles. Such that there are four task cycles, and each cycle consists of one *decision* task lasting 4 min and four *monitoring* tasks lasting 16 min in total. As each task cycle is completed, a new cycle of tasks is then scripted to begin (Figure 4) giving four complete cycles of tasks lasting 20 min each (see section NIRS Processing Pipeline for issues). Thus, the task involved maintaining stock levels in three locations, and participants were asked to make logistical decisions concerning stock allocation in order to maximize sales. Stock depletion rates were non-uniform and dependent on different demand functions. A maximum stock capacity was provided to force decisions as soon as new stock was received, and all correct, incorrect, and missed decisions were logged for later analysis.

In order to increase demand upon a participant’s level of sustained attention and task engagement, time moved at a slower pace within the simulation; this creates a monitoring task requiring a high level of sustained attention broken by brief control periods where decisions were required.

#### Experimental Conditions

For the business task, participants were randomly assigned to one of three conditions. Conditions, in this case, refer to the type and frequency of interface feedback (countermeasure) each participant received. The interface shown in Figure 3 is the default interface presented to all groups. For participants assigned to the control condition, referred to as *no interface countermeasures* (NOCM), the interface remained the same color for the duration of the task. Thus, the control group received no feedback from the interface as to their current level of sustained attention. The second condition, referred to as *continuous countermeasures* (CCM), received continuous feedback from the interface using a gradient color scheme to represent sustained attention (Figure 3). The color gradient represents a visual form of a user’s current level of sustained attention and is determined in real-time using the engagement index data, and displayed as a finely stepped, color-change surrounding the task interface. To give the user a reference, they are instructed that they will receive alerts from the interface based upon their level of sustained attention. The final condition, *event only countermeasures* (ECM), received interface feedback only when a task required interaction with the interface and if sustained attention was low; otherwise, no countermeasure was presented. Countermeasures were controlled by the engagement index (Pope et al., 1995; Mikulka et al., 2002), and were derived from EEG signals in real-time (see section Engagement Index).

#### Model of Adaptivity

**FIGURE 5:**
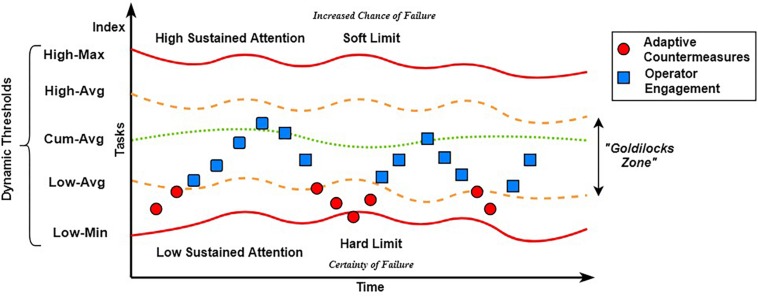
Visualized model of adaptivity, utilizing engagement index values and adaptive thresholds.

We utilized an engagement index calculated in real-time to infer the current level of sustained attention of the operator and drive the interface countermeasures as part of the BCI. Furthermore, we sought to create a model of adaptation (Figure 5), which encourages a graceful elevation and degradation of a user’s level of sustained attention (SA). This model of adaptivity was based upon work by Hockey (1997, 2011) concerning the motivational control theory of cognitive fatigue, and the cognitive energetical framework for compensatory control in the regulation of human performance under stress and high workload. The goal was not to promote and sustain a maximal level of SA within the user, but rather to promote a more fluid and dynamic “Goldilocks zone” in which SA is neither too high, nor too low to interfere with task performance. We reasoned that a low level of SA would lead to a certainty of failure through signal detection errors and that maintaining a high level of SA over a long duration would lead to an increased chance of failure due to cognitive fatigue.

With the definition of the upper and lower bounds within our model of adaptivity and with the goal of reducing the volatility of the engagement index (Ewing et al., 2016), we decided upon a series of thresholds that can be recomputed in real-time (see section Engagement Index) as the user performed the task. In Figure 5, these thresholds represent a dynamic spectrum of values ranging from the lowest–low, average–low, the mean, average–high and highest–high of the engagement index. These thresholds dynamically adjust in real-time to take into account any psychophysiological habituation that may occur over time, and then become hinge points suitable for classification, driving the interface countermeasure to adapt in real-time to promote an altered state of sustained attention. In their work, Hockey (2011) highlighted that, in general, when cognitive activities are self-motivated, and particularly when they are regarded as ‘play’, cognition does not appear to give rise to high levels of fatigue. Given the interactive and adaptive nature of the visual feedback of the interface countermeasure, we posit that any changes in sustained attention which remain within the Goldilocks zone will be due to self-directed motivational control of cognitive resources, through a ‘play’ aspect. That is, when subjects enjoy what they are doing it is no longer “workload.”

#### Engagement Index

We utilized an engagement index (Pope et al., 1995; Mikulka et al., 2002) to provide real-time assessment of a user’s level of sustained attention and to drive the neurofeedback mechanism (countermeasure) of the simulation task interface. EEG signals were recorded and utilized in real-time using NeuroRT (Mensia Technologies, Paris). Band power in the α, β and θ bands for all sites (see section Data Acquisition) was derived and divided by the total power across all electrodes to create a ratio value for each frequency band. The engagement index was then calculated as the ratio β/(α + θ). The index value was then passed to a custom controller to adapt the interface as described above, where the gradient color change is tied to both the current value of the engagement index and the adaptive thresholds; this allows the gradient to “breath”; slowly increasing and decreasing with the current level of sustained attention without changing colors sharply.

#### Classification

**FIGURE 6:**
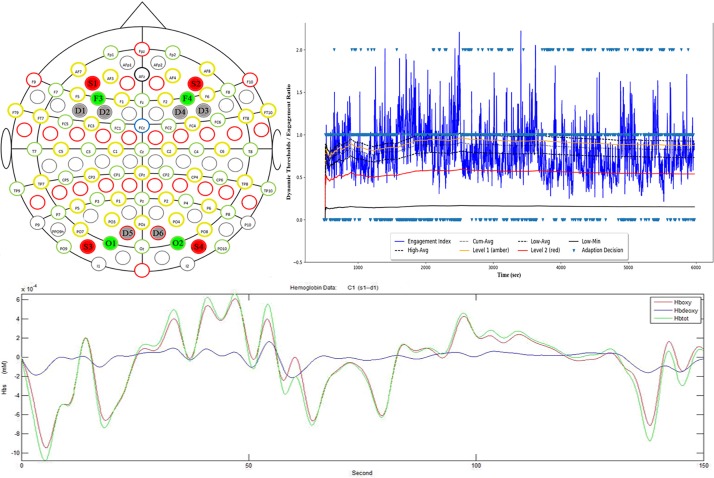
**(Upper Right)** EEG electrode and fNIRs optode placement; **(Upper Left)** real-time BCI input showing, engagement index values and adaptive thresholds; **(Bottom)** example fNIRs signal acquired from Source 1 Detector 1 Frontal Region.

The task engagement metric is not without its drawbacks as a driver of real-time adaptive behaviors within a closed biocybernetic loop or BCI, having proven unstable when used within adaptive games (Ewing et al., 2016; Labonte-Lemoyne et al., 2018). To address these drawbacks a framework was developed (Demazure et al., 2019) which utilizes the engagement index within a computational classifier using a series of adaptive dynamic thresholds derived during the calibration phase of the experimental protocol. The index value thresholds (high maximum, high average, total average, low average and the low minimum values of the index values, see Figure 6) are computed as several cumulative averages directly after the calibration task to create a personalized spectrum of values representing task engagement. This produces a cumulative average:

$$C\,A_{n+1}=\frac{x_{n+1}+\sum_{i=1}^{n}x_{i}}{n+1}$$

And for the lower/upper bound:

$$L\,o\,w\,A\,v\,e\,r\,a\,g\,e_{n+1}=C\,A_{n+1}.\left[\frac{\bar{l}}{\left(\frac{\sum_{i=1}^{n}\left(h_{i}+l_{i}\right)}{\left(n_{h}+n_{l}\right)}\right)}\right]$$

Where *CA*_*n*_is the current cumulative average of the engagement index calculated during calibration, *x*_*n*_ is a new real-time index value, and *l* and *h* are the low and high engagement index value samples gathered during calibration.

Once defined these thresholds are recomputed in real-time using a sliding window of 5 s every 1 s. Classification decisions are made based upon 5 s of historical index values compared to the current threshold values allowing the classifier to respond correctly to changes in the engagement index due to psychophysiological factors over time. Two levels of attention are classified (with a logic switched third state) – low and high sustained attention – which drives the neurofeedback mechanism and thus the interface countermeasure (see Figure 6), such that low sustained attention are shown as critical (red), losing focus (amber), and focused (white). These levels are computed as ratios (see following equations) in real-time and multiplied by the current cumulative average:

$$l\,e\,v\,e\,l\,1=\frac{1}{2}\,\left[\bar{l}+\frac{\sum_{i=1}^{n}\left(h_{i}+l_{i}\right)}{\left(n_{h}+n_{l}\right)}\right]$$

$$l\,e\,v\,e\,l\,2=\frac{1}{2}\,\left(\bar{l}-m\,i\,n\,\left(l\right)\right)$$

Where *l* is the sample collected during calibration representing low sustained attention and *h* the equivalent for high sustained attention.

### Data Acquisition

A 32-electrode EEG montage (Brainvision, Morrisville, NC, United States) was utilized to measure variations in brainwave activity in the θ (4–7 Hz), α (8–12 Hz) and β (13–21 Hz) frequency bands, from frontal and occipital cortical regions at the F3, F4, O1, O2 sites on the international 10–20 system (Jasper, 1958). These data were captured at 500 Hz and down sampled to 250 Hz in real-time. Data were filtered in real-time using a low pass filter of 1 hz, a high pass filter of 50 z, notch filter set to 60 hz. Artifacts were detected utilizing a Riemannian Potato (Barachant et al., 2013) automatic and adaptive detection method, and artifacts were flagged within the BCI architecture; if long-duration artifacts were detected the BCI shut down, data segments for short-duration artifacts were imputed to allow the BCI to continue operation. After signal down-sampling, the power bands (θ, α, β) and the engagement index were computed using a 0.5 s moving window with no overlap. A vector was created consisting of the power band values, ratio, and artifact flag, then pushed to BCI. A moving average is calculated for the previous 5 s; every second, and a classification decision is then made based on the current ratio compared to the adaptive thresholds every 5 s or every 10 data points.

NIRs data were recorded concurrently at 15 Hz using an 8 × 8 channel NIRSport device (NIRx, Berlin) from sites adjacent to F3, F4, O1, O2 (see Figure 6) using a custom six-channel montage. Channels covering the frontal positions F3-F4 consisted of 1 source and 2 detectors, and channels covering occipital regions O1-O2 channel consisted of 1 source and 1 detector. The coordinates for the NIRS sensors were chosen to both capture the effect at the primary sites and to correspond with the average path length of the emitted near infra-red light which has been reported to follow a “banana-shaped” or ellipsoid path with an approximate head penetration of 2–3 cm (Haeussinger et al., 2011). Using the extended 10–20 system as a reference for optode placement to estimate the cortical regions underlying those optodes was demonstrated by Singh et al. (2005).

#### Subjective Workload Assessment

We used a short version of the NASA-TLX (Hart, 2006), the RAW-TLX, without the additional weighting questions, for quickly assessing the perceived aspects of mental workload *post hoc* versus the full NASA-TLX. This questionnaire combines six factors: mental demand, physical demand, temporal demand, task performance, frustration level, and effort, to create a measure of overall perceived task workload.

### Analysis

#### NIRS Processing Pipeline

NIRS data were analyzed and processed using the NIRS AnalyzIR Toolbox (Santosa et al., 2018). The data were downsampled to 5Hz and then filtered using a wavelet filter with a sym8 basis function to remove motion artifacts and low-frequency characteristics (Molavi and Dumont, 2012). Data were then converted to optical density using the modified beer-lambert law (Jacques, 2013) applied with a partial path length factor of 0.1 (3 cm), giving values for oxygenated hemoglobin (HbO) and deoxygenated hemoglobin (HbR). A statistical model was generated via a mixed-effects general linear model, implementing an iteratively reweighted least-squares approach (GLM AR-IRLS) as described in Barker et al. (2013) and Santosa et al. (2018). This model outputs statistically significant effects associated with the region of interest (ROI) covered by the NIRS probe, effect type (Response) as oxygenated (HbO) or deoxygenated (HbR) hemoglobin (see section General Linear Model fNIRS Analysis), and type of task being performed, such as decision or monitoring by block, i.e., cycle 1, decision 1, monitoring 1. Some data were lost due to signal acquisition failure, unrecoverable artifacts or participants departing the experiment due to discomfort. Thus, the final monitoring cycle consists of only 9 min of data rather than 16 min in order to generate a consistent statistical model.

#### Wavelet Coherence

Wavelet coherence analysis also referred to as wavelet transform coherence (WTC), is a tool for analyzing localized variations of power within a time series, or deriving the cross-correlation between two time series signals as a function of frequency and time, potentially uncovering phase-locked behavior (see Torrence and Compo, 1998 for a practical guide). WTC has been used successfully in several studies in a wide variety of fields (Murphy et al., 2009), including assessing prefrontal connectivity using NIRS (Han et al., 2014), investigating dynamic cerebral autoregulation in neonatal hypoxic-ischemic encephalopathy (Tian et al., 2016), and for hyperscanning to reveal increased interpersonal coherence in the superior frontal cortex during cooperative tasks (Cui et al., 2012).

For the current study, WTC is used to assess the relationship (as significant coherent activity) between the NIRS signal and engagement index generated by each participant in each group (CCM, ECM, NOCM). That is, do changes in the engagement index driven by the feedback mechanism - as part of a closed-loop - show concomitant changes in the hemodynamic response indicative of increased brain activity for a given period? Wavelet coherence was performed in MATLAB (2017) (Wavelet Toolbox 4.1.9, MathWorks Inc, Natick, MA, United States) using custom scripts to derive data sets for each participant and group. Each dataset consisted of the per-one-second engagement index value and the channel aggregate average per one second of NIRS (HbO) data for each participant for the duration of phase 2 of the experiment, to obtain the wavelet coherence as a function of time and frequency. This form of analysis allows an observation of how the BCI, which utilizes the engagement index to drive interface interactions and user behavior, affects brain activity as measured by fNIRS.

Typically a wavelet coherence analysis outputs a magnitude squared coherence graph, which displays the frequency of the coherent activity in Hz on the *y*-axis and time on the *x*-axis. Coherent activity between the two signals is then plotted according to its confidence interval, where brighter shading represents higher confidence. Thus, larger, brighter areas represent more significant coherent activity in both the time and frequency domain. This form of analysis output is used in the pairwise comparisons in the following sections (Pairwise Comparisons CCM Coherence Results, Pairwise Comparisons ECM Coherence Results, Pairwise Comparisons NOCM Coherence Results) as both a narrative form and quantitatively.

To further increase the rigor of the wavelet coherence analysis, further statistical tests were performed to these data. To perform the wavelet coherence analysis, the first 10 min (600 s) of business task (phase 2) data -corresponding to the task preamble- was removed. Then for each participant within each group, NIRs data and the EEG data (consisting of engagement index values) were aggregated to create a dataset and a wavelet coherence analysis performed for each participant. The result from this analysis is 133 frequency bands of magnitude-squared coherence data every second between the two signal types for a total of 4500 s, which corresponds to the length of the task. Coherence data were then averaged for every 15 s of data to coincide with a typical hemodynamic response function (Scholkmann et al., 2014). To reduce the number of frequency bands for analysis, data were then separated into task types (decision and monitoring), and the 133 frequency bands averaged every 22 bands to produce a total of 6 frequency intervals: I: 0 – 0.06, II: 0.06 – 0.13, III: 0.13 – 0.4, IV: 0.2 – 0.26, V: 0.26 – 0.33, VI: 0.33 – 0.4 Hz which represents the mean magnitude squared coherence between the two signal types EEG and fNIRS.

Typically, coherence analysis is performed between two signals of the same type; i.e., NIRS signal data, which would then indicate the level of coherence as the similarity between the two signals in both the frequency and time domain. However, in this case, WTC is used to indicate how changes in one signal (EEG) drive changes in the other (fNIRS) - i.e., how the BCI and interface countermeasure influences EEG signal, which in turn influences changes in the NIRS signal, which in this case is the proxy for brain activity and energy utilization as HbO and HbR. Data were analyzed using a generalized linear mixed model (GLMM) with two-tailed *p*-values adjusted for multiple tests using the Holm-Sidak method (SAS, 2013).

## Results

### Wavelet Coherence

**FIGURE 7:**
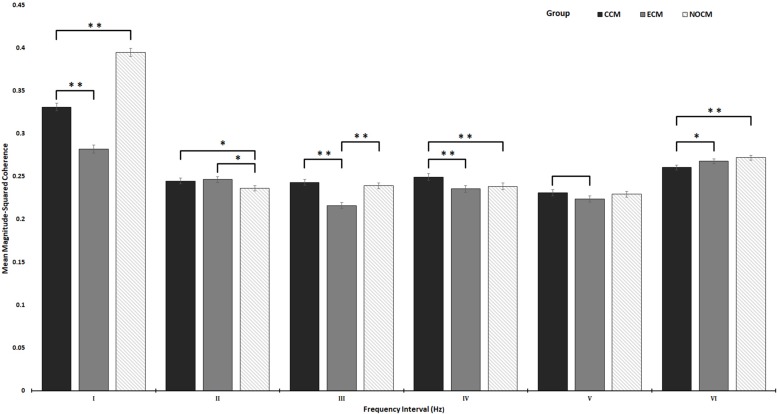
Comparison of the mean magnitude-squared coherence in the six frequency intervals between the three groups (CCM, ECM, NOCM), frequency intervals represent an equal distribution of wavelet coherence between EEG and NIRs signal data as six frequency intervals I: 0 – 0.06, II: 0.06 – 0.13, III: 0.13 – 0.4, IV: 0.2 – 0.26, V: 0.26 – 0.33, VI: 0.33 – 0.4 Hz; significant differences between the groups are marked ^∗^*p* < 0.05, ^∗∗^*p* < 0.001.

The results (Figure 7) show significant differences between the three groups with respect to the coherent activity within the defined frequency intervals, for the duration of the business task. However, this analysis does not reflect any significant differences between task cycles and removes the temporal aspect from the WTC. Due to the density of the reported results and the high number of interactions, the results report in tabular form are listed in their entirety in Supplementary Dataset S1.

Within interval I, significant differences were found in coherent activity between the three groups. The NOCM group was shown to have the highest coherent activity (*p* < 0.001) when compared to both the CCM and ECM groups. This is followed by the CCM group who had significantly more coherent activity compared with the ECM group (*p* < 0.001). Significant differences were found in interval II between both active condition groups CCM (*p* = 0.028) and ECM (*p* < 0.0073) when compared to coherent activity in the NOCM control group. No significant difference was found between the two active condition groups for this frequency interval.

Within interval III, significant differences were found between the CCM (*p* < 0.001) and the NOCM (*p* < 0.001) groups when compared with the ECM group. Additionally, while no significant difference was identified between the CCM and NOCM groups, the data points to higher activity within this frequency interval for the CCM group, possibly indicative of a trend that did not reach significance. For interval IV significant differences in coherent activity were found between the CCM group and both the ECM (*p* < 0.0001) and NOCM groups (*p* < 0.0064). No significant difference in coherence was found between the ECM and NOCM groups. Within interval V, no significant difference in coherent activity were found between any of the groups after corrections. However, before correction a difference was noted between the CCM and ECM groups [pre-correction (*p* = 0.00363); post-correction (*p* = 0.0699)]. This could potentially indicate a trend toward greater coherent activity in this frequency interval for the CCM group.

For interval VI a weak but significant difference in coherent activity between the ECM (*p* = 0.038) and the NOCM (*p* < 0.0001) when compared with the CCM group was noted. No significant difference in coherent activity between the NOCM and ECM groups was detected. Overall, this appears to indicate that both the NOCM and ECM groups displayed greater coherent activity in this lower frequency interval when compared to the CCM group.

Considering these results in the context of how the BCI potentially drives changes in both the engagement index (as a measure of sustained attention) and hemodynamic activity, which in this case is indicative of sustained attention and “on-task” engagement. Greater levels of coherent activity within intervals I – IV can be considered to indicate higher levels of brain activity and thus, greater levels of sustained attention and task engagement. Conversely, greater levels of coherent activity in frequency intervals V – VI could be considered to indicate lesser sustained attention and task engagement. Overall the results indicate, that both the continuous and control interface countermeasures groups displayed greater levels of coherent activity than the group that received interface feedback only during event phases of the task. Furthermore, the CCM group showed significantly more activity in the II, III, and IV frequency intervals when compared to the other groups.

This between-group comparison analysis has some associated limitations, utilizing mean magnitude squared coherence has smoothed the overall responses in each frequency interval for each group, reducing the peak magnitude response and removing the temporal aspects of wavelet coherence analysis.

### Pairwise Comparisons CCM Coherence Results

To characterize the relationship of coherence between the BCI and NIRS response on a within-task, between-group basis, pairwise comparisons were performed as part of the GLMM. As previously described, the experimental timeline was partitioned into decision and monitoring cycles lasting 4 and 16 min, respectively, such that there are four decision and four monitoring cycles. The full tabulated results of the pairwise comparisons are supplied in Supplementary Dataset S1.

**FIGURE 8:**
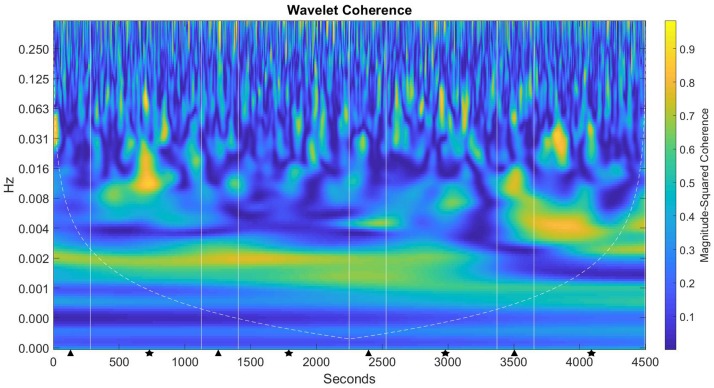
Magnitude-squared coherence plot for the CCM group. The left axis is the frequency of coherent activity in Hz; the bottom axis is time (s). Straight lines denote task cycles with decision (triangle) – monitoring (star), shaded contours represent areas of coherent activity of greater than 95% confidence.

Figure 8 shows the level of magnitude-squared coherence in both the frequency and time domain for the duration of the experimental session. In this figure, there can be seen a high degree of magnitude-squared coherence for significant portions of the experimental task (shown as lighter shading within the figure). This coherence manifests in the 0.001 – 0.008 frequencies, with additional bursts of activity between the 0.008 – 0.031 frequencies for the entire duration of the experiment. These frequency zones correspond with frequency intervals I – IV from the statistical analysis described above. In this case, the lower the interval, the higher the frequency, which then indicates potentially higher brain activity during those task cycles.

Results for the CCM group show that during the first decision task cycle, significantly higher coherence activity was present in frequency interval IV when compared to both the ECM (*p* < 0.0001) and the NOCM (*p* < 0.0001) groups. As the experimental timeline progressed, significant coherent activity was seen during the first monitoring cycle in frequency interval I (*p* < 0.0001) compared with the ECM group, and in interval II compared to both the ECM (*p* = 0.0138) and NOCM (*p* = 0.0178) groups, respectively. In the second decision cycle, significant activity is seen in frequency interval I (*p* = 0.032) compared with the ECM group, and in interval V compared with both the ECM (*p* < 0.001) and NOCM (*p* = 0.0428) groups respectively. During the second monitoring cycle, significant coherent activity was found in intervals I (*p* < 0.000), III (*p* = 0.012), and IV (*p* = 0.02) when compared with the ECM group. During the third decision cycle, significant coherent activity was found in interval I (*p* = 0.0168) compared with the ECM group, interval II (*p* = 0.044) compared with the NOCM group and intervals IV (*p* = 0.0043) and V (*p* = 0.0011) compared with the ECM group.

### Pairwise Comparisons ECM Coherence Results

**FIGURE 9:**
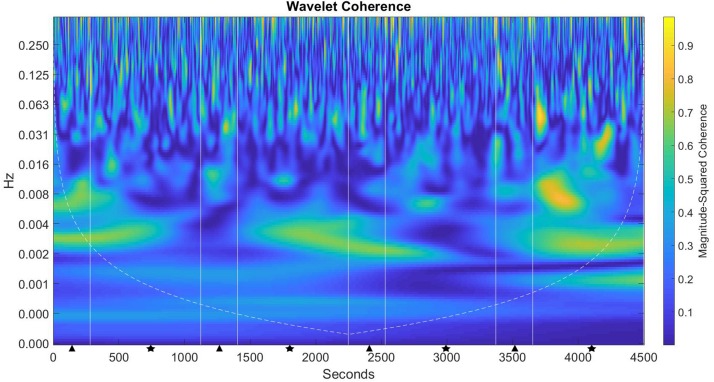
Magnitude-squared coherence plot for the ECM group. The left axis is the frequency of coherent activity in Hz; the bottom axis is time (s). Straight lines denote task cycles with decision (triangle) – monitoring (star), shaded contours represent areas of coherent activity of greater than 95% confidence.

For the event synchronized countermeasures group, pairwise comparisons showed no significant coherence activity when compared to either the CCM or NOCM groups. The previously reported significant activity within interval II does not present within any specific task cycle event in these comparisons. Figure 9 shows sporadic coherent activity, in decision cycles 1 and 2 and during all monitoring cycles within the 0.002 – 0.004 Hz frequencies. However, this activity failed to reach significance when compared to either the CCM or NOCM groups in these tests. A tentative conclusion could be drawn that the activity of the BCI.

### Pairwise Comparisons NOCM Coherence Results

Finally, the no-countermeasures control group showed significantly higher coherent activity in interval II (*p* < 0.0001) when compared with the ECM group during the first decision cycle. During the first monitoring cycle, significant coherent activity was seen in interval III (*p* < 0.001) and interval V (*p* = 0.0119) when compared with the CCM and ECM groups respectively. During the second decision cycle, significant activity was identified in interval II (*p* = 0.005) when compared to the CCM group. During the third decision and monitoring cycles of the task, significant activity was seen in intervals I (*p* = 0.001) and VI (*p* = 0.006). For the final decision cycle, significant activity was seen in interval I (*p* = 0.01) and III (*p* < 0.0001) when compared to the CCM and ECM groups respectively and in intervals V (*p* = 0.001) and VI (*p* = 0.02) when compared to the CCM group. Significant activity was also seen in the final monitoring cycle in intervals I (*p* = 0.003) and V (*p* = 0.019) compared with the CCM group.

**FIGURE 10:**
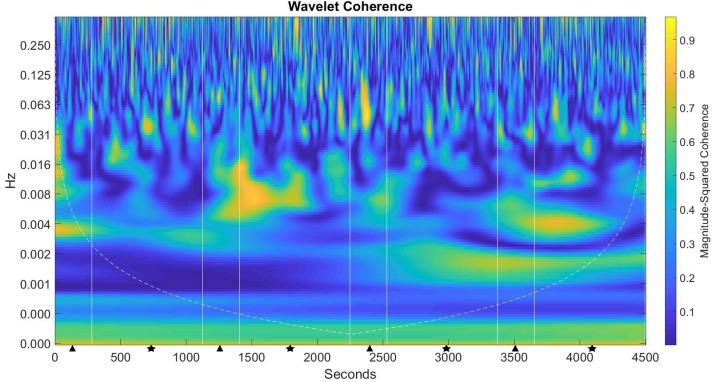
Magnitude-squared coherence plot for the NOCM group. The left axis is the frequency of coherent activity in Hz; the bottom axis is time (s). Straight lines denote task cycles with decision (triangle) – monitoring (star), shaded contours represent areas of coherent activity of greater than 95% confidence.

Figure 10 shows no consistent long duration coherent activity in the 0.001 – 0.004 frequency range, but rather bursts of coherent activity throughout. Perhaps significant for this group is the sporadic high-intensity short duration bursts of activity in the 0.004 – 0.031 frequency range. This is consistent with the pairwise comparisons, which showed predominant activity in intervals III–VI, and these bursts appear higher in number than either of the groups that received some form of interface countermeasure. Task activity, in this case, appears reduced overall when compared to the other two groups, perhaps showing evidence of a decrease in sustained attention. However, it should be noted that while activity appears to be of lesser intensity, there is still significant activity during each task cycle.

The results from the wavelet coherence analysis indicate that there are significant interactions between the BCI and potential modulation of the user’s level of sustained attention as expressed by the magnitude-squared coherence between both the EEG signal and fNIRS response. Both the CCM who received interface feedback continuously and the NOCM control group that received no interface feedback display similar levels of significant activity overall. However, activity for the CCM group is concentrated in the higher frequency intervals I – IV, which indicate greater brain activity, compared to the NOCM group, which showed greater activity in the lower frequency intervals III–VI.

### General Linear Model fNIRS Analysis

Hemodynamic activity was recorded concurrently throughout the experiment with a twofold purpose, firstly to ascertain if fNIRS would prove a suitable measure as input for a hybrid BCI and secondly to allow a more direct measurement of brain activity during the completion of the task. As the recording of fNIRS is decoupled from the operation of BCI, in that its output is not used as input for the BCI, the data remains unaffected except as a measure of “driven” brain activity from which energy utilization can be inferred.

As discussed previously, the GLM analysis was performed using the NIRS AnalyzIR Toolbox on a per task block basis comparing group by task type. Data were partitioned into one decision cycle followed by one monitoring cycle for each task block. The results, Benjamini–Hochberg FDR-corrected (Benjamini and Hochberg, 1995) for the first task block (see Table 1), indicate that for the first decision cycle, both the ECM and NOCM groups display the most significant hemodynamic activity (HbO), and that this activity was located in the left and right frontal regions. Although the data indicate higher activity in frontal regions for the ECM group, the effect did not reach significance and contrasting the ECM, and NOCM results showed no significant difference in activity. Contrasting the ECM and CCM results showed significant activity [β = 25.248, *SE* = 12.779, df(105), *t* = (1.9757), *p* < 0.05] and [β = 41.184, *SE* = 18.837, df (105), *t* = 2.1863, *p* < 0.03] for left and right in frontal regions respectively for the ECM group. During the monitoring cycle, significant activity was observed in the left occipital region for the CCM group; when this activity was contrasted with the NOCM group the activity remained significant [β = −46.736, *SE* = 22.76, df (105), *t* = −2.0534, *p* < 0.04]. However, when contrasted with the ECM group no significant difference was observed, indicating similar levels of hemodynamic activity. The general model indicated further significant activity during the monitoring cycle for both the ECM and NOCM groups, located in both the left- and right-frontal and occipital regions for the ECM group and in the left- and right-frontal regions for the NOCM group. When the frontal activity for these groups was contrasted, the general activity model became non-significant for the ECM group but remained significant for the NOCM group. Contrasting the occipital activity for these groups showed that the effect remained strong for the ECM group [β = 68.995, *SE* = 17.349, df (105), *t* = 3.9769 *p* < 0.001].

**TABLE 1:** General linear model results for entire NIRS probe for the first task block.

**ROI**	**Response**	**Contrast**	**Beta**	***SE***	**DF**	***t***	***p***
F3	HbO	Decision 1 Group ECM	35.2668	8.133786	105	4.335841	<0.0001
F4	HbO	Decision 1 Group ECM	28.27645	6.631214	105	4.264144	<0.0001
O1	HbO	Decision 1 Group NOCM	34.2609	13.35627	105	2.565155	0.011726
O1	HbO	Monitoring 1 Group CCM	44.47294	17.54123	105	2.535338	0.012708
O1	HbO	Monitoring 1 Group ECM	–38.1025	12.93245	105	–2.94627	0.003962
O2	HbO	Monitoring 1 Group ECM	–66.4483	16.30014	105	–4.07655	<0.0001
F3	HbO	Monitoring 1 Group ECM	–25.3084	8.109508	105	–3.12083	0.002329
F4	HbO	Monitoring 1 Group ECM	–28.7054	6.614271	105	–4.33992	<0.0001
F3	HbO	Monitoring 1 Group NOCM	–26.0549	7.138458	105	–3.64994	0.000411
F4	HbO	Monitoring 1 Group NOCM	–14.8185	4.903691	105	–3.0219	0.003156

Overall for this decision-monitoring cycle, it would appear that the CCM and NOCM groups displayed similar levels of hemodynamic activity, with the ECM group displaying the greatest level of hemodynamic activity. This potentially indicates that this group expended more effort to stay task-orientated, or an effect of the interface intervention for this group. Moving forward in time (first 20 min) to the monitoring cycle, it appears that the CCM group is more highly task-engaged. Extrapolating from the contrasts and the general model, it appears that by the end of the task block the NOCM group are expending more effort to maintain a state of sustained attention conducive to completing the task.

In the second task block, the overall hemodynamic activity (Table 2) showed that the CCM and NOCM groups displayed significant activity (both HbO and HbR) in both frontal and occipital regions during the decision and monitoring cycles. However, this activity was predominantly observed in the NOCM group. However, when contrasting the NOCM group with both the CCM and ECM groups for the decision cycle, the effects observed from the general activity model, for HbO and HbR in the right occipital region become non-significant. A significant effect was observed for HbR in the left frontal region for the NOCM group [β = −13.742, *SE* = 5.6311, df (84), *t* = 2.4404, *p* < 0.016774]. Contrasting the NOCM and CCM groups for the monitoring cycle shows a significant HbR response in the right frontal region for the CCM group [β = 6.7841, *SE* = 3.2148 df (84), *t* = −2.1103, *p* < 0.037811]. Additionally, after contrasting the NOCM, CCM and ECM groups, the significant HbO activity for the NOCM group observed in the general model, becomes non-significant. However, contrasting the ECM and CCM groups for this monitoring cycle shows a strong HbR effect for both channels of the left frontal region, [β = 9.3662, *SE* = 4.0584, df (84), *t* = 2.3079, *p* < 0.023464] and [β = 14.69, *SE* = 6.95, df (84), *t* = 2.1137, *p* < 0.037508] respectively for channels 2–3, 2–4 of the NIRS probe.

**TABLE 2:** General linear model results for entire NIRS probe for the second task block.

**ROI**	**Response**	**Contrast**	**Beta**	***SE***	**DF**	***t***	***p***
O2	HbR	Decision 2 Group CCM	–12.8867	6.098971	84	–2.11293	0.037575
O2	HbO	Decision 2 Group NOCM	20.57677	9.101398	84	2.260837	0.026355
O2	HbR	Decision 2 Group NOCM	–13.0064	3.574234	84	–3.63893	<0.0001
F4	HbO	Monitoring 2 Group NOCM	–10.3252	3.727819	84	–2.76977	0.006903
F3	HbR	Monitoring 2 Group CCM	–7.45334	2.998012	84	–2.4861	0.014896
O2	HbO	Monitoring 2 Group NOCM	18.42991	9.101392	84	2.024955	0.046048

While it is not customary to report HbR activity, the response was strong enough throughout this task block to warrant mention, as this response coupled with the lack of a significant direct two-phase neurovascular coupling response is potentially interesting. A possible interpretation could be that we are observing some form of temporal cycling hemodynamic effect, in which the length of time spent on-task by participants could have resulted in a significant “flushing” of oxygenated blood to remove metabolic by-products. Conversely, it could be that the NIRS probe design is not granular enough to record the full gamut of hemodynamic activity. Overall the GLM results indicate that both the CCM and NOCM groups were active (in terms of HbO) during this task block, and that in general the NOCM group were more engaged during this task cycle, indicating higher metabolic activity (interpreted as “mental effort”) to maintain sustained attention while “on-task.” However, the overall decrease in hemodynamic activity highlights that a potential decrement in sustained attention and task engagement was manifest for all groups.

The GLM results for the third task block (Table 3) showed no significant differences in hemodynamic activity was observed between the groups for the decision cycle. However, significant activity was observed during the monitoring cycle in the right-frontal and right-occipital for the NOCM and CCM groups, respectively. Contrasting NOCM and CCM responses for this cycle showed a clear coupled neurovascular response for the NOCM group, with weak but still significant HbO activity [β = 17.337, *SE* = 8.4743, df (76), *t* = −2.0459, *p* < 0.044229] and strong HbR activity [β = −10.533, *SE* = 3.9263, df (76), *t* = −2.6828, *p* < 0.01]. Contrasting the CCM and ECM groups shows the observed right occipital activity remains significant [β = 33.999, *SE* = 15.127, df (76), *t* = 2.2475, *p* < 0.027], indicating that in this instance, right occipital activity was more prevalent for the CCM group than the ECM group and non-significant when compared to activity from the NOCM group. No other significant hemodynamic activity was observed for this task block. A possible interpretation for the lack of overall significant activity for this task block could be that a decrement in sustained attention is well established by this point (circa 56 min of task time). However, both the CCM and NOCM groups still display some significant activity during the monitoring cycle.

**TABLE 3:** General linear model results for entire NIRS probe for the third task block.

**ROI**	**Response**	**Contrast**	**Beta**	***SE***	**DF**	***t***	***p***
O2	HbO	Monitoring 3 Group CCM	–23.6298	11.01096	76	–2.14602	0.035063
F4	HbO	Monitoring 3 Group NOCM	–6.93781	2.755174	76	–2.5181	0.013902

For the final task block, the GLM results showed no significant activity other than a significant HbR response for the CCM group [β = 24.54102, *SE* = 11.24811, df (52), *t* = 2.181791, *p* < 0.033667] in the right occipital region during the monitoring cycle. This response once contrasted with the two other groups became non-significant. It is a fair assumption, that by this time a decrement in sustained attention and task engagement is firmly in effect, with only the CCM group showing minimal but still significant activity.

### Engagement Index

Given that the BCI and interface feedback countermeasure is driven by the engagement index, statistical analyses were performed to determine if a significant difference exists between groups and between task cycle types, i.e., decision or monitoring. To test this, we performed a one-way analysis of variance (ANOVA) and found a significant statistical difference between the three conditions, ECM - NOCM and CCM - NOCM [*F*(2,2297) = 71.78, *p* < 0.001]. The results showing the NOCM group with a significantly lower level of SA when compared to the other two groups. However, no significant statistical difference was found between the CCM and ECM (*p* = N.S.) groups. Furthermore, a two-way ANOVA reported a weak but still significant difference in the level of sustained attention between the decision and monitoring cycles [*F*(1,2177) = 5.72, *p* < 0.05] for all groups.

### NASA Task Load Index

To measure perceived metrics of workload and performance we employed the simplified RAW-TLX version of the NASA Task Load Index (NASA-TLX). The Raw-TLX Scores, mean raw, and subscales are shown in Table 4. by condition. The subjective perceived workload is the average of the six subscales: mental demand, physical demand, temporal demand, performance, effort, and frustration, scored on a twenty-step bipolar scale. The condition with CCM shows the lowest total score with 7.27 (σ = 3.1). The highest score comes from the event-related countermeasures (ECM) who reported a surprisingly high level of frustration and lesser self-reported performance.

**TABLE 4:** NASA-TLX mean (σ) scores for each condition.

**Condition**	**CCM**	**ECM**	**NOCM**
Raw TLX	7.27 (3.1)	9.7 (3.3)	9.2 (3.4)
Mental Demand	9 (5.7)	13.6 (6)	12.8 (5.7)
Physical Demand	5.5 (6.4)	2.9 (3.8)	3.9 (3.96)
Temporal Demand	5.2 (5.1)	3.3 (4.6)	6.5 (7.2)
Performance	7.8 (2.9)	13.7 (5.1)	8.9 (3.4)
Effort	7.5 (4.9)	10.4 (5.3)	11.4 (5.7)
Frustration	8.8 (4.4)	15.2 (5.8)	11.6 (4.8)

We found no statistical difference between the Raw TLX, or the subscales via ANOVA, with the exception of self-perceived performance [*F*(2,20) = 4,305, *p* < 0.028], which asks the question “*How successful do you think you were in accomplishing the goals of the task set by the experimenter (or yourself)? How satisfied were you with your performance in accomplishing these goals*?” Here there was a significant difference between ECM and CCM conditions was reported, with the ECM group reporting a perception of higher performance.

### Performance Metrics

#### Sales and Estimated Missed Sales

To drive the behavior of the participant during the business task, the participant was asked to maximize sales. To measure performance, two metrics were created: total sales and estimated missed sales. The CCM group had the best performance with an average of 7.46% (σ = 1.76) of estimated missed sales, and mean total sales of 14,785 (σ = 423), compared with 14,180 (σ = 875), 9.62% (σ = 4.91) and 14,529 (σ = 510), 9.79% (σ = 2.75) for ECM and NOCM groups respectively. However, no significant statistical difference in performance metrics was observed when comparing the conditions via ANOVA.

While differences in performance in terms of sales and missed sales appear moderate, they are still apparent with a trend of increased sales and decreased errors. Given that the task was designed for long durations, potentially if the task was run for a longer period, the margin between the groups may well have widened. This aspect is one for further study.

#### Actions per Minute

To calculate the amount of activity each participant spent interacting with the interface, we created a metric: actions per minute (APM) to determine if the countermeasures had any effect on the number of user actions during task completion. We observed a significant statistical difference between the conditions, for ECM – NOCM and CCM – NOCM [*F*(2,2297) = 12.05, *p* < 0.001], but no significant difference between ECM – CCM. For the entire duration of the simulation, the CCM and ECM conditions had a higher mean APM of 3.460 (*SE* = 0.140) and 3.317 (*SE* = 0.139) respectively. The NOCM group displayed a lower APM with 2.65 (*SE* = 0.097). There was an observed gradual rise in APM during the decision cycles compared to the monitoring cycles, with the CCM group spending more time interacting with the interface at these times when compared to the two other groups, and on average the CCM group spent more time interacting with the interface during monitoring periods.

## Discussion

### Theoretical Basis

Theoretical approaches and experimental findings concerning mental effort are often assumed to be a natural consequence of the demands of a task (Egeth and Kahneman, 1975). However, Hockey (2011) proposed that effort should be considered as an optional response to the awareness and assessment of task demands under the control of the individual. In their view, it is the adoption of high effort responses to task demands which drive the fatigue process, rather than the presence of demands themselves. They provide examples from the literature that found the greater vigilance decrement associated with higher effort requirements was accompanied by increased subjective fatigue. They further expand upon this by adding the element of controllability as a moderator of the workload-fatigue relationship, in which controllability refers to an individual’s perception that they have control over work activities. They confirmed this moderating effect in an experimental study of office work, in which workload was manipulated by time pressure and opportunity to schedule tasks (Hockey and Earle, 2006). In the present study, interpreting the GLM results in the context of the coherence analysis highlights that for the NOCM group, the bursts of coherent activity appear associated to some extent with the significant activity reported by the GLM. Potentially this indicates higher energy utilization through a need to sustain attention, which was not moderated by interface countermeasures. However, for both the CCM and ECM groups, this association is not as closely coupled as would be expected from the coherence analysis. That is, periods of coherent activity do not have a corresponding significant hemodynamic effect reported by the NIRS GLM. Thus, it would appear that significant overall coherent activity over long durations did not equate to significant hemodynamic responses in this instance, which appears to be counter-intuitive.

### Interpretation

Interpreting the results using this proposition provides a more intuitive explanation of the effects observed within the current study. The NOCM group (who received no interface feedback countermeasures) were reliant on internal motivational mechanisms to sustain attention and perform the task, resulting in sporadic bursts of high effort and thus greater hemodynamic activity. The results from the coherence analysis appear to provide some evidence to support this, in that the magnitude-squared coherence plot (Figure 10) shows bursts of brain activity during key points in the task timeline. The results from the GLM appear to loosely associate with significant hemodynamic activity during those same periods. Furthermore, the results from the subjective TLX inventory indicated that participants perceived much the same thing, by self-assessing higher perceived effort, frustration, mental and temporal demand than either of the other two groups. However, from the performance metrics, sales and actions per minute, the NOCM group spent less time interacting with the interface [mean APM 2.65 (${\text{σ}}_{\bar{x}}$ = 0.097)]; yet still performed better than the ECM group in terms of sales with 14,180 (σ = 875) average sales and 9.62% (σ = 4.91) average missed sales, compared to 14,529 (σ = 510) and 9.79% (σ = 2.75).

In the ECM group, the magnitude-squared coherence plot (Figure 9) shows a pattern of coherent activity that appears to follow the event procedures that spawn the interface countermeasure, i.e., activity when task cycles start and are dependent on the level of sustained attention. There were, however, significant areas of no activity where the interface countermeasure appears to have been ineffective, which could be evidence of a signal miss error. Moreover, while there were areas where the interface countermeasure drove behavior -evidenced by increased coherent activity, overall, there was no statistically significant hemodynamic response. This effect appears to conform to earlier work concerning the vigilance decrement and signal detection errors (e.g., Parasuraman et al., 1996; Miller and Parasuraman, 2003; De Boer and Dekker, 2017). Such that, even with the interface countermeasure(s) in place, participants were still required to maintain a sufficient level of sustained attention in between events in order to detect the signal. The analysis of the subjective assessment of workload, in this case, appears to confirm this, with the highest overall TLX score and participants in the ECM group reporting the highest mental demand, effort and frustration, lowest physical and temporal demand and yet self-assessing the highest task performance. The performance metrics do not support this self-assessed level of task performance, with the ECM group performing worst overall for total sales and average missed sales, yet interacting more with the interface in terms of mean APM 3.317 (*SE* = 0.139) than the NOCM group 2.65 (*SE* = 0.097).

For the CCM group who received continuous interface countermeasures, the magnitude-squared coherence plot (Figure 8) shows a pattern of consistent coherence activity throughout the duration of the task, with only brief periods of low coherence activity. However, this pattern of coherence activity was not reflected in the GLM results for this group which showed very little significant hemodynamic effects after the first decision-monitoring cycle. A possible explanation for this may be found in the effort-control mechanism proposed by Hockey (1997). The CCM group were given continuous feedback as to their current level of sustained attention; we suggest that this information was utilized to self-regulate effort and cognitive resource allocation for the entire duration of the task. Moreover, the interface feedback mechanism potentially added a supplementary element of perceived control, in which the use of the color gradient allowed participants to modulate their level of engagement with the task in a way that was neither “too high” nor “too low.” Modulating these two factors may have served to augment or bypass the neural mechanisms proposed in the cognitive energetic framework of compensatory control, in that the ability to self-modulate effort (and thus cognitive resources) required less neural “hardware” leading to greater neural efficiency when performing the task.

The results for the CCM group appear to provide some evidence to support these assertions. Using the hemodynamic response function as a proxy for the mobilization and metabolism of cognitive resources; both the level of engagement and the hemodynamic responses showed the most consistent coherence; this coupled with a lack of significant hemodynamic responses as reported by the GLM, highlights potential cognitive efficiency gains through lesser energy utilization. In terms of performance metrics, the CCM group had the best task performance with higher mean total sales 14,785 (σ = 423) and the lowest average of missed total sales 7.46% (σ = 1.76) when compared to the other two groups; this group also interacted more often with the interface in terms of actions per minute 3.460 (*SE* = 0.140) when compared to the other two groups. However, there was no significant difference between the CCM and ECM groups in terms of APM; what does appear to be noteworthy is the effect of each action upon overall task performance, in what appears to be a moderate net positive effect of both quantity and quality of interactions with the interface. The analysis of the subjective assessment of workload shows the CCM group with the lowest mean TLX score, indicative of a perceived lower workload overall.

Furthermore, the CCM group also recorded the lowest scores in all factors that make up this test, i.e., mental demand, effort and frustration, physical and temporal demand, and concluding with an overall assessment of low task performance. This perception of low task performance appears incongruent considering the moderate performance improvement compared to the ECM and NOCM groups. However, allowing for an apparent inverse relationship between the measured neurophysiological responses, and the self-assessment of all factors consisting of perceived workload; it is not hard to imagine that since participants were in actuality expending less effort to perform the task. From this, they would conclude that they performed worse at the task; being unaware of the beneficial effect of augmenting their ability to self-modulate sustained attention.

The overall aim of this research was to develop and test a brain-computer interface which allowed the user to modulate their level of sustained attention by utilizing the engagement index as a measure of sustained attention and on-task engagement. A simple neurofeedback mechanism (driven by the BCI) was provided in real-time to combat a decrement in sustained attention. The results for this study provide some evidence to support our hypothesis that a simple continuous interface feedback mechanism, in the form of a color gradient representing low to high sustained attention, would allow users of the system to self-regulate their sustained attention within “optimal” boundaries as defined in our model of adaptation. The results also provide support for a narrative in which users of the BCI which provided continuous interface feedback, utilized less cognitive “energetical” resources as evidenced through both objective and subjective measurements, leading to moderate improvements in task performance and a decrease in on-task errors. Moreover, users of the BCI which only provided interface countermeasures when synchronized to events and their current level of sustained attention performed worse at the task potentially due to signal detection errors and an over-reliance on event signaling. Finally, the control group who received no interface feedback (NOCM), appeared to use more cognitive resources as measured by NIRS to perform the task, yet performed better than the ECM group who received the event-driven interface feedback, and worse than the CCM group who received continuous feedback.

### Limitations

A sub-goal of our development of the BCI was to investigate the use and efficacy of fNIRS with regards to its integration into the BCI architecture. However, in this instance, we found that fNIRS, given the type and duration of the task, did not prove as useful a tool as expected. Some participants reported high levels of discomfort, leading them to depart the experiment before completion. We also experienced a high degree of signal loss or artifacts within the recorded signal showing it not to be as robust as first supposed from the literature (e.g., Maior et al., 2015; Lukanov et al., 2016). However, this could be an effect of our experimental procedure and our probe design rather than a fundamental problem with the technology. Furthermore, the shape of the human head and the fact certain hair and skin types preclude its use or add unwanted artifacts to the data make fNIRS problematic for ecologically valid work tasks in real-world scenarios, and concomitantly for real-time BCI applications. However, we do understand that NIRS, as with all technologies, is undergoing an accelerated evolution in terms of size, comfort and reliability and that potentially within 5 years the technology will be unrecognizable by today’s standards and ready to be applied in contexts requiring real-time feedback.

Another limiting factor within the current study pertains to the design of the NIRS probe. The probe was inspired by the current literature relating to the engagement index, and thus we set out to measure hemodynamic responses from those sites. Another limitation is that of sample size, with the previously discussed issues with data reduction due to discomfort, signal loss or artifacts, our eventual sample size reduces the positive impact of the results reported here. However, we believe that the results we report here give a good indication of how much stronger the effect sizes would have been, given larger sample sizes.

### Final Remarks

The future of human labor appears to be heading in the direction of significant RPA, and this will change how humans interact with technology, creating new types of task. These new tasks, at least those that include a human-in-the-loop, will require an optimum level of sustained attention and involve short decision and long monitoring cycles. It would appear, at least based upon the results presented in this study, that some form of continuous feedback mechanism to ensure a sufficient level of sustained attention is maintained. This type of feedback could prove beneficial to the workers of tomorrow becoming an important tool in human-machine teaming and more generally for increasing task performance in the coming decade, as technology advances to the point where external to physiology monitoring technologies are no longer the cutting edge.

## Conclusion

The overarching goal of this study was to test an online BCI that utilizes an engagement index, a novel adaptive dynamic threshold classification method and a simple neurofeedback mechanism. The BCI was developed to allow a user to modulate their level of sustained attention while performing a long duration business logistics task. Both EEG and fNIRS were utilized in the BCI architecture, EEG to provide the engagement index and fNIRS to monitor the responses and provide data for *post hoc* analysis. Wavelet coherence analysis was used to illustrate how the BCI and feedback mechanism drove user behavior, using fNIRS as a proxy for energy utilization. The results of this study provide evidence to support a hypothesis that “utilizing a BCI to allow the self-regulation of sustained attention, can have beneficial effects upon operator task engagement and task performance.”

Wavelet coherence analysis revealed patterns of user activity consistent with our hypothesis in that the continuous interface countermeasure group (CCM) spent the most time interacting with the interface followed by the synchronized to task events countermeasure group (ECM) and then the control group for which the BCI was recording data but inactive. Using actions per minute (APM) as a metric for interface activity we found that the CCM and ECM groups had significantly higher APM [*F*(2,2297) = 12.05, *p* < 0.001] when compared with the control NOCM group. The combination of Wavelet coherence analysis and general linear model applied to the fNIRS data revealed that while the CCM group spent more time interacting with the interface, they appeared to expend less effort to complete the 90-min task, followed by the ECM and then NOCM groups who expended significantly more effort to complete the task in comparison.

Analysis of the metrics of perceived workload and performance measured using the simplified RAW-TLX version of the NASA Task Load Index (NASA-TLX) appear to support this from a user point of view. The CCM had the lowest total score with 7.27 (σ = 3.1) when compared with the ECM 9.7 (σ = 3.3) and NOCM 9.2 (σ = 3.4) groups. However, a one-way between-groups ANOVA showed no statistical difference (*p* > 0.5) between the Raw TLX or the subscales with the exception of self-perceived performance [*F*(2,20) = 4,305, *p* = 0.028] when comparing the CCM and ECM groups.

In terms of task performance metrics the results show that when the BCI was used to deliver a continuous interface countermeasure, task performance was moderately enhanced in terms of total and estimated missed sales 14,785 (σ = 423), 7.46% (σ = 1.76), when compared to both the control group were the BCI was not active (NOCM) 14,529 (σ = 510), 9.79% (σ = 2.75), and the group which received countermeasures and dependent on their current level of sustained attention (ECM), 14,180 (σ = 875), 9.62% (σ = 4.91).

While differences in performance in terms of sales and missed sales appear moderate, they are still apparent and create a net positive of increased performance in terms of higher sales and fewer errors, higher levels of interface interactivity (time on task) and less energy utilization (regulated sustained attention). Future studies will focus on the integration of fNIRS response features and increasing both duration of the task and applying the BCI in a different context, such as a safety-critical task where the ability to self-modulate sustained attention could potentially lead to significant reductions in signal error detection in turn leading to enhanced on-task safety.

## Ethics Statement

This study was carried out in accordance with the recommendations of the Tri-Council Policy Statement: Ethical Conduct for Research Involving Humans, HEC Research Ethics Board (REB) with written informed consent from all subjects. All subjects gave written informed consent in accordance with the Declaration of Helsinki. The protocol was approved by the ‘HEC, Research Ethics Board (REB).’

## Author Contributions

AK: ideation, coordination, design and development, experimental procedures, analysis, interpretation, and writing. TD: ideation, design and development, implementation, architecture, and analysis. P-ML: supervision, insight, and editing ideation. EL-L: ideation, technical problem solving, and editing. SS: ideation, insight, and supervision. MF: insight and ideation. GB: ideation, insight, and supervision.

## Conflict of Interest

The authors declare that the research was conducted in the absence of any commercial or financial relationships that could be construed as a potential conflict of interest.
